# Late presentation of fractures of the lateral condyle of the humerus in children

**DOI:** 10.4103/0019-5413.67119

**Published:** 2011

**Authors:** Shyam K Saraf, Ghanshyam N Khare

**Affiliations:** Department of Orthopedics, Institute of Medical Sciences, Banaras Hindu University, Varanasi, India

**Keywords:** Elbow injuries, lateral condyle fracture, late presentation of lateral condyle fractures, children

## Abstract

**Background:**

The current controversy regarding the management of fractures of the lateral condyle of the humerus presenting between 3 to 12 weeks prompted us to evaluate our results of open reduction and internal fixation of such fractures.

**Patients and Methods:**

Twenty-one patients operated between March 1995 and February 2001 qualified for this study. Five patients presented between 3–4 weeks, nine between 5–8 weeks and seven between 9–12 weeks post injury. Ten fractures were classified as stage II and eleven as stage III (Jacob *et al*. criteria). The mean age was 8 years (range: 4–14 years). All patients underwent surgery (open reduction and internal fixation with K-wires/screw, with or without bone grafting). The results were assessed by the modified criteria of Agarwal *et al*. after an average follow-up of 2.3 years.

**Results:**

Excellent to good results were observed in all the five patients presenting at 3–4 weeks post injury. In the patients presenting at 5–8 weeks, the results were excellent in one, good in four, fair in three, and poor in one patient. The fracture united in all cases; however, malunion was observed in four patients. The fractures that were operated at 9–12 weeks showed good results in one case, fair result in three cases, and poor result in three cases. Avascular necrosis of the lateral condyle in one patient, premature fusion in two patients, pin tract infection in three patients, and gross restriction of elbow movements in three patients were the major complications in this group. Accurate reduction was difficult as a result of new bone formation and remodeling at the fracture surfaces. Multiple incisions over the common extensor aponeurosis and bone graft supplementation were helpful for achieving acceptable reduction.

**Conclusion:**

Open reduction and internal fixation is recommended in all cases of displaced fractures of the lateral condyle of the humerus presenting at up to 12 weeks post injury. However, the results become poorer with increase in duration after injury and the grade of displacement. To avoid complications it is important to carry out careful dissection of the soft tissue attachments and to mobilize the fragment without the use of force.

## INTRODUCTION

Fractures of the lateral condyle of the humerus constitute around 13%–18% of elbow injuries, with the peak occurring at the age of 6–7 years. The management of fresh displaced fractures of less than 3 weeks duration is not controversial as it is generally agreed that it should be treated by osteosynthetic procedures.^1^–^3^ Although there could be some difference of opinion regarding the approach, fixation method (wire *vs* screw), or period of immobilization,^4^–^6^ etc., the consensus remains in favor of operative intervention. The problem arises when the patient presents late due to socioeconomic reasons, lack of awareness, missed diagnosis, or improper initial treatment. It has been observed that nonunion and growth arrest more commonly result from minimally displaced fractures than from markedly displaced and rotated fractures, probably because severe fractures are treated more adequately with surgery.^7^ A late presentation leads to difficulty in management due to displacement of the fragment as a result of the pull of the common extensors, incongruous reduction of articular surfaces, injury/early closure of the epiphyseal growth plate, and possible damage to vascular supply because of stripping of soft tissue attachments. For these reasons, when the patient presents at 3–12 weeks, the controversy is with regard to whether to treat these fractures by nonoperative or operative methods. If these fractures are treated nonoperatively, the various possible complications are nonunion, malunion, deformity at the site, instability of the elbow joint, stiffness, cubitus valgus/varus, and tardy ulnar nerve palsy. On the other hand, when these fractures are treated operatively, there could be peroperative problems of reduction. In addition, precarious blood supply to the fractured fragment due to excessive stripping of the soft tissues, may result in avascular necrosis of the fragment.^3^,^8^ So the majority favor management of established nonunion by no treatment as the functional problems are not very severe.^3^,^9^ It is easier to treat cubitus valgus/varus at a later date by corrective osteotomy or to treat tardy ulnar nerve palsy by ulnar nerve transposition rather than to attempt a difficult reduction. Despite the inherent risk associated with the surgery, there are reports in the literature of successful outcomes of open reduction and internal fixation of these established nonunion cases.^10^–^12^ Against this backdrop of controversy regarding the management of cases presenting at 3–12 weeks post injury, we report our experience of treating a series of 21 such cases.

## PATIENTS AND METHODS

This retrospective study included 21 patients of fracture of the lateral condyle of the humerus presenting at 3–12 weeks post injury who were treated by open reduction and internal fixation from March 1995 and February 2001. All the patients presented with limitation of elbow movements and lateral prominence at the elbow. Pain was the next most common complaint (16/21). As almost all presented with some degree of fixed flexion deformity of the elbow, it was not possible to assess the cubitus varus/valgus deformity correctly at the time of presentation. 12 patients had been treated elsewhere with above-elbow plaster-of-Paris (POP) cast, with or without manipulation and nine of the patients had no history of treatment (other than oral medicines). Plain radiographs (anteroposterior and lateral) of both the elbows were obtained. The informed consent for surgery was obtained. The patients were operated upon by one of the authors.

### Operative procedure

A lateral approach was used^13^. A gentle dissection of the fracture fragment was done, with minimum stripping of the soft tissue attachments on it. Multiple incisions over the fascia of the common extensors were placed in an attempt to close the gap between the fragments.^14^ We avoided the use of sharp instruments to force the reduction. The curettage of the sclerosed fracture end of the distal humerus was done. In those cases presenting with gross restriction of movements and of less than 6 weeks’ duration, gentle force was applied with the aim of increasing the range of flexion/extension motion to some extent. Anatomical reduction was attempted in every case; however, it was not possible in fractures more than 6 weeks old, and in these cases we accepted the best possible reduction. In some of the older fractures, in order to get acceptable reduction, careful curettage of new bone/callus was required. In five patients, a bone graft taken from the iliac crest/ulna was used. Whenever possible, depending upon the size of the metaphysis in the fracture fragment, we used the cancellous screw for fracture fixation (six cases). When it was not possible to pass the screw without damaging the physis, we used plain K-wires (minimum two in number). Postoperatively, an above-elbow POP slab was applied for about 6–9 weeks, depending upon the status of the union. The wires were removed between 5–7 weeks, but the POP slab was continued. Mobilization exercises of the elbow were started after removal of the slab. For the assessment of results, the patients were evaluated clinically for pain, range of motion, carrying angle, and deformity at the local site (i.e., lateral prominence). The radiological points noted during evaluation were reduction, status of the growth plate, evidence of avascular necrosis of the fractured fragment, congruity of the joint, status of union, and deformity. The criteria as defined by Aggarwal *et al*.^15^ were modified a little and followed; i.e.:

*Excellent*: Union in perfect alignment, full range of elbow movement, no alteration in carrying angle, no premature fusion of physis, no avascular necrosis of epiphysis, no lateral prominence, and X-ray showing anatomical reduction.

*Good*: Union with minimum displacement, limitation of terminal range of movements of not more than 15°, no alteration in carrying angle, no premature fusion of physis, no avascular necrosis of epiphysis, no deformity at local site, and X-ray showing step/gap of not more than 2 mm.

*Fair*: Union with minimum displacement, limitation of terminal range of movements of up to 25°, alteration in carrying angle of up to 10°, premature fusion of the physis, no avascular necrosis of epiphysis, mild deformity at local site, and X-ray showing a step/gap of between 2–5 mm.

*Poor*: Nonunion at fracture site, gross limitations of elbow movements (with limitation more than 30°), change in carrying angle of more than 10°, premature fusion of the physis, avascular necrosis of the fragment, visible deformity at local site, and X-ray showing a step/gap of more than 5 mm.

## RESULTS

**Figure 1:**
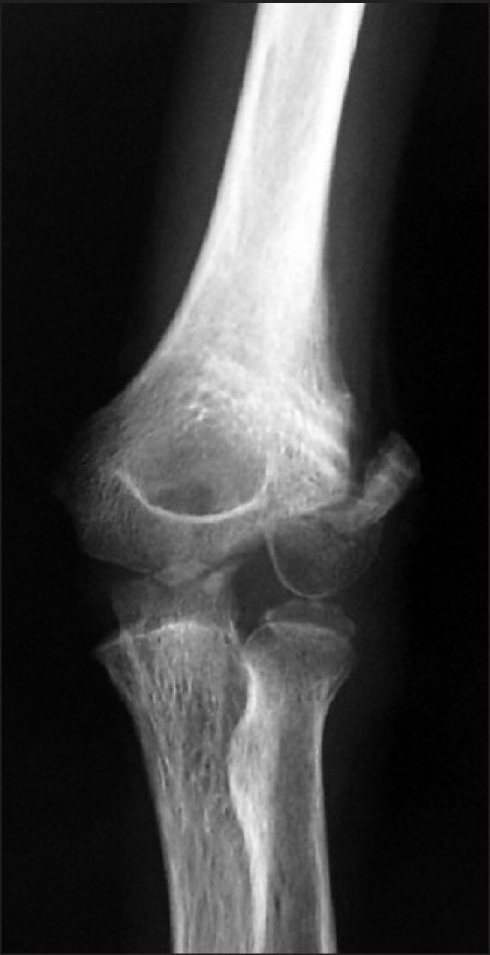
X-ray of the elbow joint (anteroposterior view) showing loss of reduction due to premature removal of K-wires

**Figure 2:**
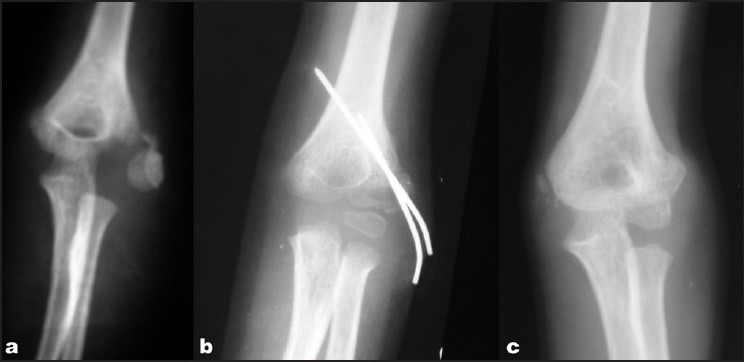
X-ray of the elbow joint (anteroposterior view) showing (a) eight-week-old fracture of the lateral condyle of the humerus. (b) Immediate postoperative X-ray after fixation with two K-wires; (c) same patient after union

**Figure 3:**
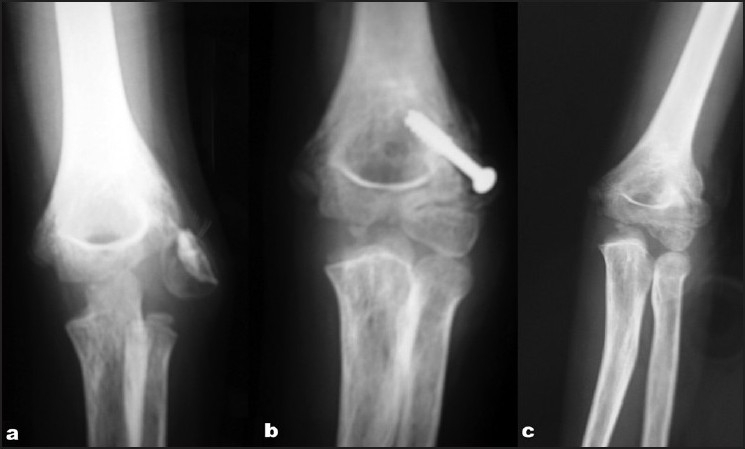
X-ray of the elbow joint (anteroposterior view) showing (a) ten week old fracture of the lateral condyle of the humerus. (b) Less than satisfactory reduction at 8 week follow up, (c) 20 week follow up

**Figure 4:**
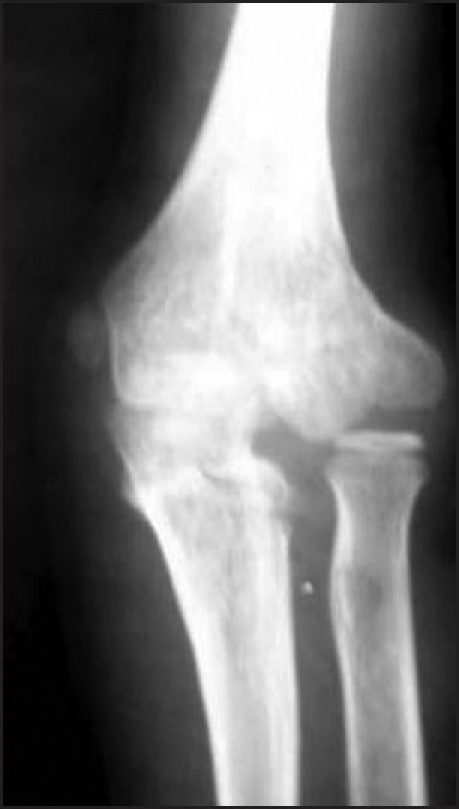
X-ray of the elbow joint (anteroposterior view) at 8 week follow up showing premature closure of the epiphysis and fishtail deformity

The average age at the time of surgery was 8 years (range 4–14 years). Eighteen were Milch type II fractures and three were Milch type I. Five patients presented between 3–4 weeks, nine between 5–8 weeks, and seven presented between 9–12 weeks post injury [Table 1]. The follow-up period ranged from 15 months to 4 years, with an average follow-up duration of 2 years and 3 months. Nine patients presented to us without having received any treatment elsewhere, whereas the other 12 patients had a plaster cast applied elsewhere before they came to us. As per Jacob *et al*.classification,^3^ ten were classified as stage II and eleven as stage III. Bone graft was required in five patients, and in seven patients multiple incisions were made in the aponeurosis of the common extensors. On outcome rating as per Aggarwal *et al*. criteria, five patients had excellent result, six had good result, six had fair result, and four had poor result. On clinical evaluation, none of our patients had any preoperative or postoperative signs of ulnar nerve involvement. One patient developed severe postoperative pin tract infection; the relatively early removal of K-wires at 4 weeks in this case led to loss of reduction [Figure 1]. Two other patients developed mild pin tract infection; the wires were removed at 5 weeks in both the cases. Significant increase (by 40°–80°) in the total range of flexion/extension movements at final follow-up was noticed in 16 patients; in 3 patients the improvement was between 20°–25°, and in the other 2 patients the improvement was less than 10° (both the cases were stage III displacement: one of 9 weeks’ duration and the other of 10 weeks’ duration). No significant increase in the range of movements was noticed in those patients in whom manipulation under anesthesia was tried before giving the incision. None of the patients, inclusive of our patient no 10, where there was loss of reduction due to early removal of wire as a result of deep infection showed any deterioration in the preoperative range of motion. On radiology, the fracture had united in all the cases [Figure 2 a–c]. Reduction was excellent to good in nine, fair in another nine, and less than satisfactory in three [Figure 3 a–c]; in these three patients, a lateral prominence was evident both clinically and radiologically. The average time for union was 8 weeks. Premature closure of the epiphysis was observed in two cases [Figure 4]. The results were better in those who were operated upon within 8 weeks of injury [Table 2] as well as in those presenting with lesser degree of displacement of the fragment [Table 3].

**Table 1:** Clinical details of patients

Case no.	Age (in years)/Sex	Delay between injury and treatment (in weeks)	Stage of displacement (Jacob *et al*.^3^)	Operative procedure	Total restriction of arc of motion (flexion/extention in degrees)	Comments	Result	Follow-up period (in months)
1.	5/M	3	II	OR+IF	Nil		Excellent	15
2.	11/F	8	III	OR+IF+BG	15		Good	24
3.	6/M	4	II	OR+IF	10		Good	36
4.	5/M	9	III	OR+IF	30	Premature fusion, gross restriction of movements, cubitus valgus 12°	Poor	32
5.	14/M	7	II	OR+IF	Nil		Excellent	20
6.	7/F	11	II	OR+IF+BG+Z plasty	20	Superficial pin tract infection	Fair	22
7.	4/M	7	II	OR+IF	10		Good	40
8.	9/F	10	III	OR+IF+Z plasty	40	Gross restriction of movements	Poor	30
9.	8/M	4	II	OR+IF	Nil		Excellent	48
10.	12/M	5	II	OR+IF	18	Premature removal of wires due to deep pin tract infection, leading to loss of reduction	Fair	18
11.	6/M	8	III	OR+IF+Z plasty	15		Good	32
12.	6/M	8	III	OR+IF+Z plasty	25	Premature fusion, cubitus valgus 10°	Poor	27
13.	7/F	3	II	OR+IF	Nil		Excellent	33
14.	8/F	6	III	OR+IF+BG+Z plasty	18	Cubitus varus	Fair	24
15.	9/M	10	III	OR+IF	15		Good	18
16.	8/M	11	III	OR+IF+BG+Z plasty	22		Fair	20
17.	9/M	4	II	OR+IF	Nil		Excellent	26
18.	8/F	7	III	OR+IF	20	Superficial pin tract infection	Fair	36
19.	10/F	6	II	OR+IF	10		Good	37
20.	5/M	10	III	OR+IF+BG+Z plasty	25	Prominence at distal lateral humerus	Fair	22
21.	6/M	9	III	OR+IF	45	AVN, gross restriction of movements	Poor	20

**Table 2:** Results according to delay between injury and operative procedure

Delay between injury and surgery (in weeks	Number of patients	Excellent	Good	Fair	Poor
3–4	5	4	1	0	0
5–8	9	1	4	3	1
9–12	7	0	1	3	3
Total	21	5	6	6	4

**Table 3:** Results according to stage of displacement (Jacob *et al*.^3^)

	Number of patients	Excellent	Good	Fair	Poor
Stage II	10	5	3	2	0
Stage III	11	0	3	4	4
Total	21	5	6	6	4

## DISCUSSION

**Figure 5:**
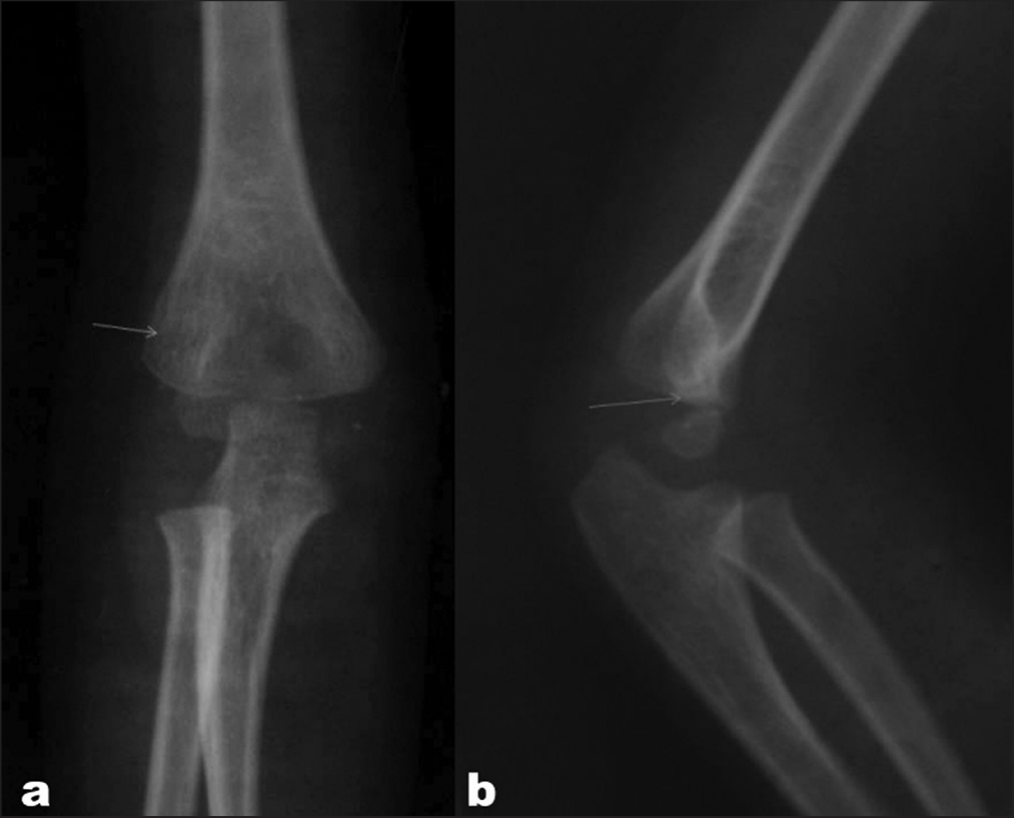
(a) Fracture in anteroposterior view of the elbow can be missed. (b) Lateral view of the elbow showing displacement of the fragment

In developing countries, patients with fractures of the lateral condyle of the humerus usually present late.^16^ Sometimes the diagnosis is missed due to incorrect interpretation of the radiograph, as the fracture fragment is partially cartilaginous; the radiographs are also often of poor quality [Figure 5 ab]. A prospective cohort study showed that internal oblique radiographs are more sensitive than a plain anteroposterior (AP) view for diagnosing displaced or minimally displaced fractures.^17^ Recently, a 20° tilt AP radiograph has been suggested to demonstrate fragment dislocation more precisely than a standard radiograph.^18^ High-resolution ultrasonography^19^ or MRI^20^ can also demonstrate the cartilage hinge and the displacement; however, these facilities may not be available in the rural and suburban areas in most developing countries. The diagnosis of minimally displaced fractures is therefore often missed in the early stages, being made late or only after more displacement has occurred.

The management of fractures of the lateral condyle of the humerus in patients presenting late remains controversial. When these fractures present 12 weeks post injury, the majority opinion is in favor of nonoperative management in order to avoid the problems of stiffness of the elbow, avascular necrosis of the fragment, and difficulty in reduction.^3^,^8^,^21^ Achieving anatomical reduction is often not possible because of remodeling of the fragment, new bone formation, and sclerosis and smoothening of the fracture line. For these various reasons, in long-standing untreated nonunion, reduction of the fracture has been a concern. With higher grades of displacement, it sometimes becomes impossible to bring the fragment into normal position without stripping the soft tissue attachments on the displaced fragment. As extensive soft tissue dissection may lead to avascular necrosis of the fragment, many recommend that these fractures should be left alone.^3^,^9^ Dhillon *et al*.^22^ do not recommend osteosynthetic procedures even after 6 weeks. Despite the disappointing results and the general disapproval of surgery, there are several reports in the recent literature in favor of surgery.^23^,^24^ Mazurek and Skorupski^11^ operated a 7-year-old boy with nonunion of 1-year duration using an olecranon osteotomy approach, with open reduction, bone grafting, and K-wire fixation, and reported excellent result at 6 months. In the series by Shen *et al*.,^25^ 13 patients with fracture of more than 4 weeks’ duration (56 days on average) were treated by open reduction and internal fixation; all had improvement in range of movements and good cosmetic outcome. In the series by Shimada *et al*.,^10^ there were 16 patients with an average interval of 5 years between injury and operation; excellent results could be obtained in eight and good result in seven patients after open reduction, bone grafting, and internal fixation with K-wires. Wattenbarger *et al*.^26^ studied the effect of late open reduction of >3-week-old lateral condyle fractures in 11 children and did not find any case of avascular necrosis even though four of their cases had displacement of more than 10 mm.

The problems and the resulting complications may not be as bad when the patient presents within 12 weeks of the injury, as careful dissection and modifications in the surgical technique can provide a satisfactory reduction without compromising the blood supply. In those cases where we could not achieve the reduction without compromising blood supply with soft tissue dissection, we used the technique of making multiple incisions in the common extensor aponeurosis as described by Gaur *et al*.^14^ This was helpful for achieving reduction. Bone graft was useful in long-standing grade III fractures where despite making multiple incisions in the extensor aponeurosis the gap could not be closed. How secure the fixation is, is also an important factor that affects the overall result. It is recommended that these fractures be fixed using nonthreaded K-wires so as not to disturb the growth plate. However, in 36 of 37 fresh fractures fixed by 4-mm partially-threaded lag screws, excellent functional results were observed by Sharma *et al*.^27^ at an average follow-up of 4.8 years; mild fishtailing was observed in three cases only. Similarly, in 19 of the 20 fractures fixed by screw, the results were rated as excellent at 1-year follow-up.^28^ The fixation by metaphyseal lag screw did not cause growth disturbances unlike what happens with the commonly used but relatively unstable fixation with Kirschner wires.^4^

We preferred to fix the fractures using a 4-mm partially-threaded screw alone when the metaphyseal fragment was large enough. A supplementary K-wire was also used in two patients in whom it was felt that fixation by a single screw would not be enough to provide stability. In our opinion, fixation by screw is more secure; however, it was not possible to use screw fixation in the majority of our cases due to the disposition of the fracture line and our apprehensions regarding damage to the physis. As these fractures are a minimum of 3 weeks old, retaining the implants for at least 6 weeks is recommend since premature removal of the wire can lead to displacement of the reduction as happened in one of our cases [Figure 1].

The minimally displaced fractures with high potential for displacement should be fixed at the earliest when the surgery is technically less demanding and functional outcome is relatively more predictable. If left untreated, it can result in malunion or, more frequently, nonunion. The treatment of malunion is even more difficult and is fraught with complications. Launey *et al*.^29^ showed displacement in 5 of the 17 fractures treated by cast immobilization; four of them required surgery at a later date. In our opinion, another reason for fixing these fractures is to enable the physis to take part in the growth process of the distal humerus; otherwise, the physis will close prematurely, resulting in even more severe deformity. Better results were observed in patients with Jacob stage II when the fracture was fixed as compared to the results in patients with more severely displaced stage III fracture [Table 3]. Duration since injury, in our opinion, is the most important factor that decides the outcome. The results were better [Table 2] in fractures that were less than 8 weeks old as compared with those of longer duration.

The cases presenting with gross restriction of movements – and of less than 6 weeks’ duration – gentle force was applied under anesthesia with the aim of providing some increase in the range of motion; however, no significant increase in the range of movements was noticed in these patients and hence we do not recommend this procedure. As far the depth of the trochlear groove and fish-tail deformity is concerned, our follow-up period was not sufficiently long to study this aspect; however, in those cases where reduction was not satisfactory, some deepening of the groove and a tendency to develop the fish-tail deformity was observed.

We conclude that open reduction and internal fixation is recommended in all displaced fractures of the lateral condyle of the humerus presenting up to 12 weeks after injury; the results become poorer with increase in duration after the injury and grade of displacement.
